# Ultrasonographic Imaging for Structural Characterization of Renal Affections and Diagnosis of Associated Chronic Renal Failure in 10 Dogs

**DOI:** 10.5402/2011/901713

**Published:** 2011-12-25

**Authors:** Vijay Kumar, Adarsh Kumar, A. C. Varshney

**Affiliations:** Department of Veterinary Surgery and Radiology DGCN, College of Veterinary and Animal Sciences, CSK HP Agricultural University, Himachal Pradesh, Palampur 176062, India

## Abstract

The present study comprises of 10 dogs of either sex with primary indication of azotaemia. All the dogs were subjected to detailed clinical, haematobiochemical, urinalysis, and microbiological examination along with radiographical and ultrasonographical examination. Based on the ultrasonographic structural abnormalities, the different renal affections associated with CRF in majority of dogs were diagnosed. The different affections included “end-stage” kidneys (*n* = 4), hydronephrosis (*n* = 1), renomegaly (*n* = 1), nephritis (*n* = 1), nephrolithiasis (*n* = 1), nephrocalcinosis (*n* = 1), and renal cyst (*n* = 1). The significant ultrasonographic features in these affections included small kidneys with loss of corticomedullary demarcation (“end-stage” kidneys); increased cortical echogenicity (nephritis); dilation of the renal pelvis, separation of the central renal sinus with anechoic space, atrophy of renal medulla, (hydronephrosis); enlarged kidneys with increased overall echogenicity of renal cortex (renomegaly and associated nephritis); hyperechoic-mineralized structure with shadowing (nephrolithiasis); diffuse, small, multiple hyperechoic structures in the renal parenchyma with distal acoustic shadowing (nephrocalcinosis); small spherical intercortical anechoic structures fluid (renal cysts). In the present study, ultrasound proved to be a quick, convenient, and sensitive modality in detecting alterations in renal size and parenchymal architecture. All the dogs so diagnosed with CRF were rendered conservative medical treatment to control clinical signs of uraemia; maintain adequate fluid, electrolyte, and acid/base balance; provide adequate nutrition; minimize progression of renal failure.

## 1. Introduction

Chronic renal failure is a syndrome characterized by inability of the kidneys to perform adequately, owing to the progressive loss of function over a period of months to years. A variety of adverse influences (e.g., toxins, overdosed drugs, infectious agents, ischaemic insults, neoplasia) can damage the kidneys, either reversibly or irreversibly, to produce renal disease. However, in many cases, a specific underlying cause cannot be identified. Approximately, 75% or more diminution in functional renal mass results in compromised excretory function to the extent that a state of azotaemia develops [1, 2]. However, neither the “magnitude of azotaemia” alone can be used to determine whether the azotaemia is prerenal, renal, or postrenal nor it can be used to determine whether the disease process is acute or chronic, reversible or irreversible, and progressive or nonprogressive. The diagnostic approach in these renal affections is to localize the azotaemia. Fortunately, prerenal azotaemia can be distinguished from acute intrinsic renal failure in many patients simply by providing generous “test” fluid therapy and monitoring to see whether the azotaemia promptly and completely resolves [1]. Vigorous efforts are required to identify the underlying cause of any active renal disease which may contribute towards progression to chronic renal failure and thus for therapeutics of treatable causes. A diagnosis of CRF is usually based on a combination of compatible history, physical examination, laboratory data, and imaging studies, which play a crucial role in differentiating acute from chronic renal failure. The minimum database for renal affection includes BUN, creatinine, urinalysis, Hb, PCV, total protein, albumin, and electrolyte assay (especially potassium, sodium, total calcium, inorganic phosphate). Contrast radiographic studies, although, help to identify or rule out potentially treatable causes of CRF, but it is laborious and associated with the risk of radiographic contrast agent-induced nephropathy [1]. Ultrasonography, on the other hand is a noninvasive and quite useful for detection and characterization of renal parenchymal details. It is the technique of choice for reliable diagnosis of fluid-filled cystic lesions, renal mass lesions, hydronephrosis, and anatomic localization of uroliths (radiopaque and radiolucent). Moreover, it is a very precise way of evaluating the degree of obstruction. Renal biopsy besides establishing definitive nephropathy offers an appropriate alternative to provide important diagnostic and prognostic information especially in cases when distinction between ARF (reversible) and CRF (irreversible) is not otherwise possible [2]. Once the CRF is established, the goals of conservative medical management of CRF are to control clinical signs of uraemia; maintain adequate fluid, electrolyte, and acid/base balance; provide adequate nutrition; minimize progression of renal failure. However, because renal lesions that cause CRF are irreversible and usually progressive, so no specific therapy is curative.

## 2. Materials and Methods

10 dogs (8 males, 2 female) aged 4–13 years, weighing between 10 kg and 26 kg, presented in Teaching Veterinary Clinical Complex, COVAS, Palampur in the year 2007-2008 with primary indication of azotaemia were subjected to detailed clinical, haematobiochemical, urinalysis, and microbiological examination along with radiographical and ultrasonographical examination to narrow the differential diagnoses/establish the renal diagnosis. Ultrasound-guided renal biopsy and histopathological examination for ascertaining definitive nephropathy although being recommended in many diseased dogs could not be undertaken because of poor owner compliance. The treatment regimen in progressive CRF comprised of fluid therapy, Wepox Injection (Human erythropoietin), Anabolic steroid Injection (nandrolone), whole blood transfusion, Imferon Injection (Shreya), Haemaccel Injection (NPIL) and Livadex Injection (Agrivet), Ranitidine Injection (Zantec), antibacterials, syrup Riconia (Ranbaxy), Gel Mucaine (Wyeth), and syrup Geriforte (Himalaya).

Haematological parameters included Hb, PCV, TEC, TLC, DLC, and biochemistry-comprised plasma ALT, AST, BUN, creatinine, total bilirubin, total protein, glucose, sodium, potassium, inorganic phosphate, and total calcium using standardized procedures. Urine following transcutaneous cystocentesis was examined for presence of blood (RBC), protein, glucose, ketone bodies, pH, leucocytes (WBC), and specific gravity and colour by using URISCAN^IVD^ 10 SGL STRIP in Semiautomatic urine analyzer UROSCAN OPTIMA II. Besides, urine sediment microscopic examination was carried out for visualizing the presence of epithelial cells, crystals, RBCs, WBCs, casts, bacteria, and debris. Radiographical examination was performed using 500 mAS, 125 kVp X-ray machine whereas ultrasonography was carried out using a BPL ultrasound scanner (a gray scale, real time, B+M-mode scanner), with 3.5 MHz microconvex and 3.5 MHz curvilinear transducer. The selection of the transducer (scan head) was based on the transducer contact area and having the organ/region/lesion of interest within the focal zone of the transducer. The images were captured only after appropriate on-screen annotation.

## 3. Results

Based on the ultrasonographic structural characterization, the affections of kidney leading to chronic renal failure (CRF) in majority of dogs were diagnosed in 10 clinical cases. These affections included “end-stage” kidneys (*n* = 4), hydronephrosis (*n* = 1), renomegaly (*n* = 1), nephritis (*n* = 1), nephrolithiasis (*n* = 1), nephrocalcinosis (*n* = 1), and renal cyst (*n* = 1). Eight dogs so diagnosed with CRF, however, died during the period of therapeutic stabilization that is, after 5–25 days of presentation.

### 3.1. “End-Stage” Kidneys

**Figure 1:**
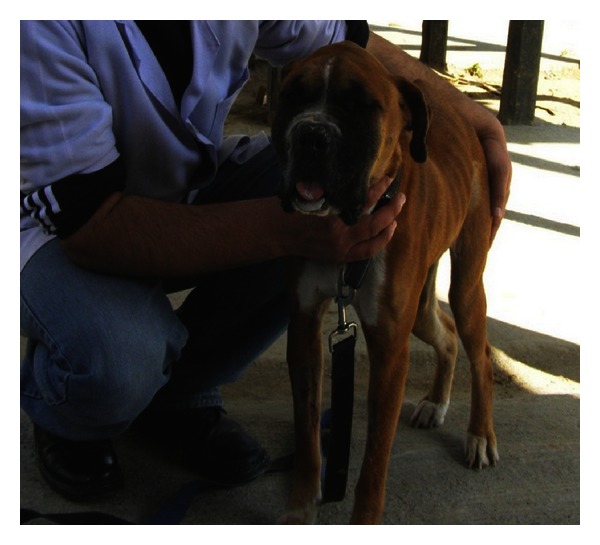
A 4-year-old, spayed, female Boxer dog with “End-stage” kidney disease showing progressive debility with markedly arched back (kyphosis) and tongue protrusion with necrosis of distal portion of tongue.

“End-stage” kidneys and associated CRF were diagnosed in four dogs (3 males, 1 female) with mean age of 8.5 years (4.0–13.0 years) and average body weight of 14.25 kg (10.0–17.0 kg). The dogs were presented with history of inappetance/anorexia, vomition, progressive lethargy, and weight loss for last 3 days to 2 months. Two dogs passed scanty and constipated faeces, whereas stool was loose in two other dogs. Oliguria was marked in three dogs. Three dogs had foul odour from the mouth. One dog which suffered with dental tarter and acute gingivitis had undergone ultrasonic dental scaling one-month before. The female dog had previously suffered with venereal granuloma and chronic endometritis. The dog was treated with chemotherapy (Inj. vincristine sulfate) 4-month before and had undergone curative ovariohysterectomy 2-month before. Physical examination revealed tucked-up abdomen in three dogs with markedly arched back in the female dog. Conjunctival mucous membrane was icteric in two dogs, whereas it was pallor in other two dogs. Glossitis with necrosis of distal portion of tongue and tongue protrusion was marked in one female dog (Figure 1). Three dogs had acidotic odour from mouth with one dog affected with severe gingivitis. These dogs evinced lumbar/renal pain on abdominal palpation.

**Figure 2:**
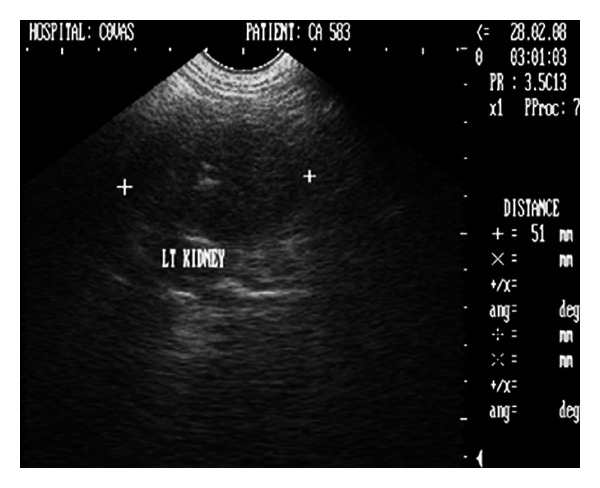
2D sonogram of left kidney in midsagittal scan revealing small-sized kidney with loss of corticomedullary definition and thus making its distinction from surrounding tissue difficult in 4-year-old, spayed, female Boxer dog.

**Figure 3:**
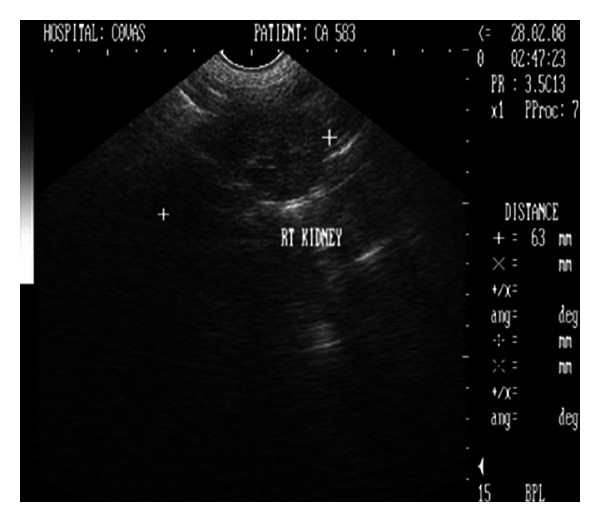
Ultrasonographic appearance (2D) of right kidney in midsagittal plane with “end-stage” kidney disease in 4-year-old, spayed, female, Boxer dog with increased medullary echogenicity.

Haematological profile (Table 1) indicated moderate-to-severe anaemia. Neutrophilic leucocytosis was detected in one dog, whereas there was significant lymphocytosis with decreased total leucocyte count and total erythrocyte count in the female dog. Biochemical profile (Table 2) indicated severe azotemia in all the dogs, whereas hyperkalaemia and hyperphosphataemia were significant in three dogs. There was marked elevation in AST activity in two dogs. Hyperbilirubinemia was marked in two dogs. The dogs passed urine with 4–6 leucocytes and 2–4 RBCs/hpf, moderate crystalluria (oxalates and triple phosphate crystals) and with specific gravity in the range of 1.007–1.012 (Table 3). Urinalysis revealed moderate proteinuria and glucosuria in two dogs. Microbiological examination of the urine in one dog yielded *Streptococcus species* with sensitivity to amikacin, gentamicin and nitrofurantoin. Survey radiographic examination could not visualize the kidneys in all the dogs. Intravenous pyelogram was also obtained in one dog, but the nephrogram and pyelogram phase could not be visualized. Ultrasonographic examination revealed typically small sized kidneys in two dogs. There was complete loss of corticomedullary definition in all the dogs. In two dogs, the renal parenchyma could hardly be differentiated from the surrounding tissue (Figures 2 and 3). All the four dogs were given treatment for progressive CRF as mentioned but died after 15–25 days of therapeutics.

### 3.2. Hydronephrosis

**Figure 4:**
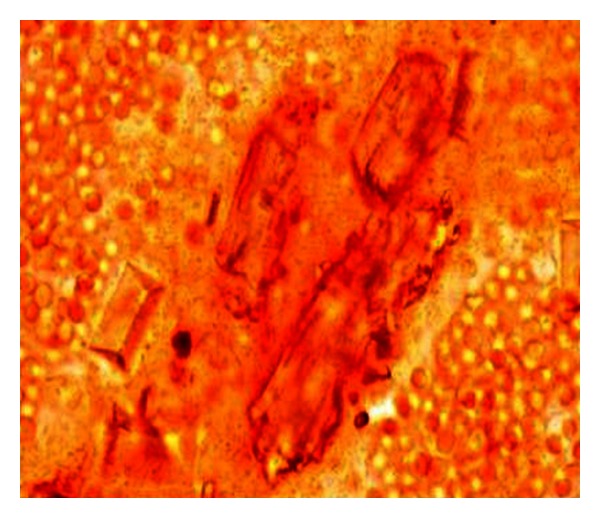
Urine sediment examination with field full of RBCs, leukocytes, and epithelial cells with occasional epithelial casts and triple phosphates crystals in a 9-year-old, intact male, Gaddi cross dog (unstained sediment; original magnification ×400).

Chronic renal failure secondary to hydronephrosis following urethrolithiasis was diagnosed in nine-year-old, intact male, Gaddi cross dog, with body weight of 22 kg. The dog was presented with a three-day history of anorexia and inappetance and vomition for the last eleven days. The dog had urine retention with infrequent blood tinged urine dribbles for the last 4 days. The dog was very lethargic and severely depressed with moderate elevation in body temperature. He had acidotic odour from mouth with severe glossitis and necrosis of distal portions of tongue. On abdominal palpation, a large and firm bladder was noted. Gentle aseptic urethral catheterization encountered resistance in pelvic urethra 2 cm (approximately) proximal to urethral opening. Following survey radiograph, urethral obstruction was relieved by propulsion of the calculi into the urinary bladder by gently advancing urethral catheter. Urinary catheterization allowed removal of approximately 1400 mL of hematuric urine. Laboratory examination demonstrated severe azotaemia, hyperkalaemia, and hyperphosphataemia. Urinalysis revealed brownish red coloured urine with specific gravity of ≥1.030 (Table 3). Urine sediment examination revealed the microscopic field filled with RBCs, leukocytes, and epithelial cells with occasional epithelial casts and triple phosphates crystals (Figure 4). On microbiological examination, no growth was observed.

**Figure 5:**
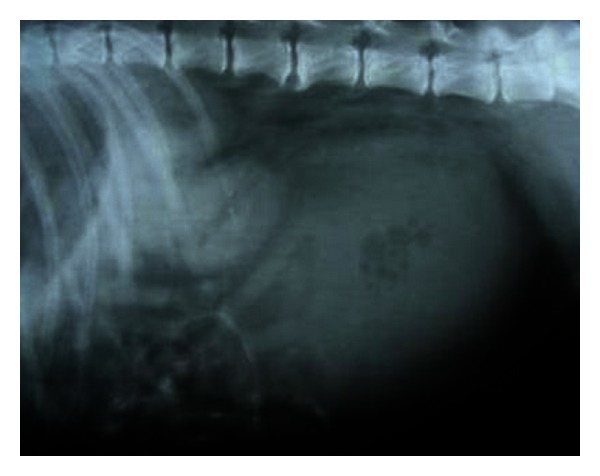
Right lateral survey radiograph with remarkable enlarged kidneys visualized following 1400 mL urine evacuation through retrohydropropulsion in a nine-year-old, intact male, Gaddi cross dog.

Survey abdominal lateral radiograph revealed severely distended urinary bladder. Partial urine removal enhanced visualization of enlarged kidneys (Figure 5). Pneumocystogram (standing lateral), following urine removal, allowed visualization of two small radiopaque calculi that were pushed into the urinary bladder by the urethral catheter.

**Figure 6:**
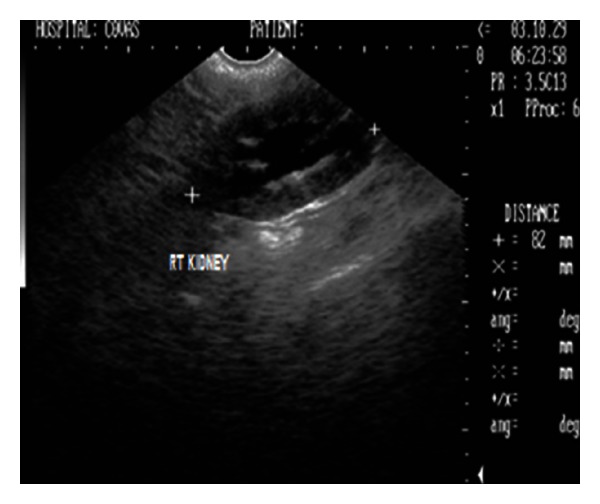
2D ultrasonogram in sagittal plane of right kidney in a 9-year-old, intact male, Gaddi cross dog revealing renal pelvic dilatation (pyelectasia) and atrophy of renal medulla.

**Figure 7:**
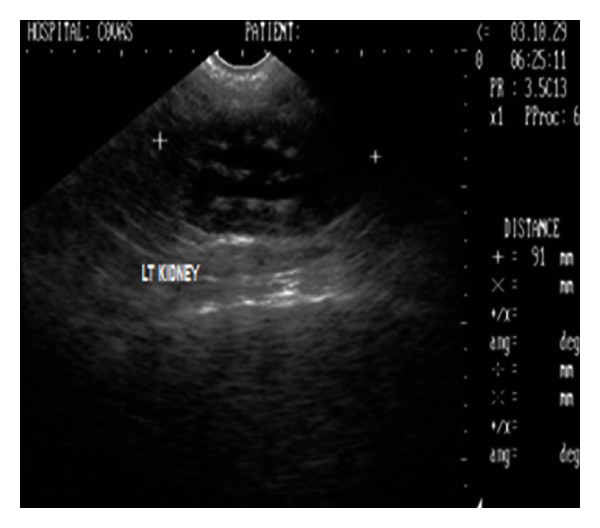
Ultrasonographic appearance (2D) of left kidney in sagittal plane showing marked renomegaly with renal pelvic dilatation and hyperechoic medullary rim in a 9-year-old, intact male, Gaddi cross dog.

Sonograph demonstrated enlarged kidneys (left and right kidneys measuring 91 mm and 82 mm in length, resp.) with renal pelvic dilation, separation of the central renal sinus with anechoic space, and atrophy of renal medulla (Figures 6 and 7). Very classic bilateral hyperechoic medullary rim suggestive of nephropathy and moderately dilated ureters were also observed. Imaging of the urinary bladder revealed hyperechoic and thickened UB wall with echogenic urine and blood clots. The dog died during the period of therapeutic stabilization.

### 3.3. Renomegaly

Renomegaly with nephritis and associated CRF were diagnosed in a two-year-old, intact male, German shepherd dog with body weight of 26 kg. The dog had 4-day history of anorexia, vomition, and oliguria. He had been gradually losing weight for the last 1.5 months. Physical examination revealed congested conjunctival mucous membrane and acidotic odour from mouth. On abdominal palpation, enlarged kidneys with mild lumbar pain were noted. The urine collected after transabdominal cystocentesis appeared grossly normal.

**Figure 8:**
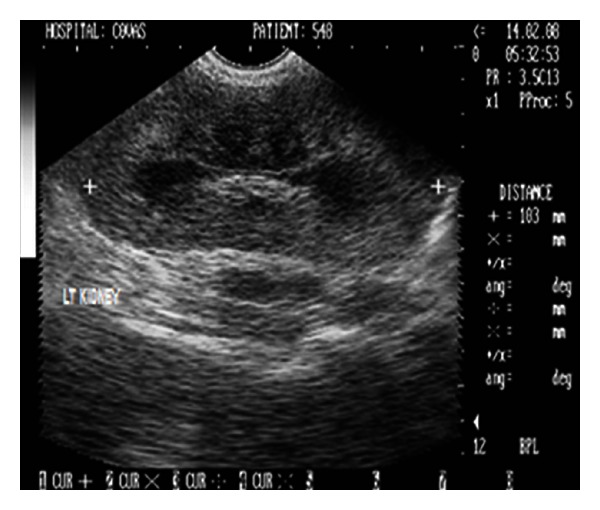
2D sonogram in midsagittal scan of left kidney in a two-year-old, intact male, German shepherd dog with ultrasonographic impression of an enlarged kidney measuring 103 mm in length and revealing increased cortical and medullary echogenicity.

**Figure 9:**
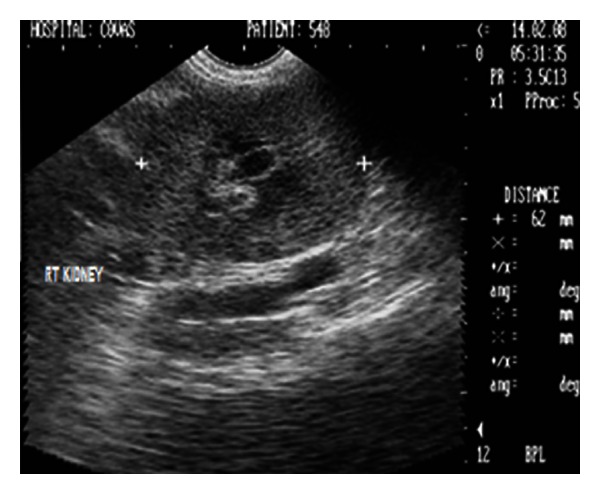
Ultrasonographic appearance (2D) in transverse scan of the enlarged right kidney with renal width measuring 62 mm and hyperechoic renal cortex in a two-year-old, intact male, German shepherd dog.

 The haematological profile (Table 1) was unremarkable whereas plasma biochemistry (Table 2) demonstrated severe azotaemia (BUN = 183.6 mg/dL, creatinine = 17.5 mg/dL) and elevated total bilirubin. Urinalysis revealed isosthenuria (urine-specific gravity: 1.012) with moderate glucosuria and proteinuria. Abdominal lateral survey radiograph did not reveal any abnormality and kidneys could not be appreciated. Sonograph demonstrated enlarged kidneys (left and right kidneys measuring 103 and 98 mm in length and 60 and 62 mm in width, resp.) with increased overall echogenicity and thickness of renal cortex. The medullary pyramids were also echogenic with poor corticomedullary definition (Figures 8 and 9). The dog although was rendered treatment, it died after 16 days of presentation in the clinics.

### 3.4. Nephritis

Chronic nephritis and associated chronic renal failure (CRF) was diagnosed in a 17-year-old, 6 kg body weight, intact female, Pomeranian dog. The dog had history of vomition and inappetance for last three days with polydipsia and polyuria for last one week. Mouth cavity examination revealed acidotic odour from mouth, dental tarter, and moderate gingivitis. Blood work (Tables 1 and 2) indicated lymphocytic leucocytosis and severe azotemia. There was increased ALT and AST activity with elevated total bilirubin. Urinalysis and urine sediment examination did not show any significant alteration except 2-3 WBCs and 2–4 RBCs/hpf with specific gravity of 1.020.

**Figure 10:**
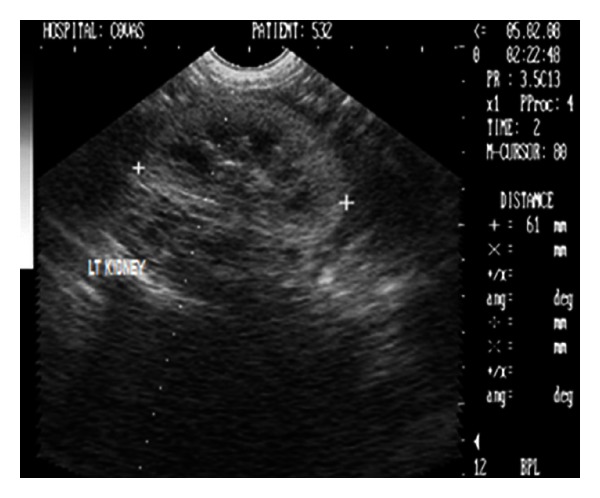
2D left renal sonogram in sagittal plane with increased cortical echogenicity and renal length of 61 mm in a 17-year-old, 6 kg body weight, intact female, Pomeranian dog.

**Figure 11:**
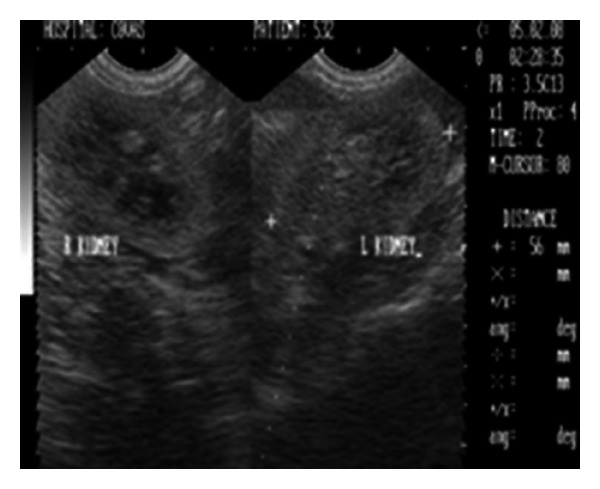
B+B sonograph of left and right kidneys with increased cortical echogenicities in a 17-year-old, intact female, Pomeranian dog.

 Radiographic examination was unremarkable. Ultrasonographic examination revealed bilateral markedly increased echogenicity of the renal cortex comparable with spleen (Figures 10 and 11). Corticomedullary junction was however fairly defined. Gall bladder was distended and hepatic parenchyma was hypoechoic with occasional hyperechoic foci. The dog was treated with antibacterials, antiemetics, inj. Ranitidine, and fluid therapy. The dog died after 25 days of presentation in the clinics.

### 3.5. Nephrolithiasis

CRF following nephrolithiasis (bilateral) with associated nephritis and cystolithiasis was diagnosed in a 9-year-old, intact male, Doberman pinscher dog with body weight of 18 kg. The dog had 4-day history of anorexia and vomition. Physical examination revealed lumbar pain on abdominal palpation. Haemato-biochemical findings (Tables 1 and 2) revealed mild neutrophilic leucocytosis and severe azotemia (BUN-128 mg/dL, creatinine 5.4 mg/dL). Increased ALT and AST activity with hyperbilirubinemia was also noticed. Urinalysis was unremarkable except 6–8 RBCs, and 4–6 WBCs/hpf, crystalluria and isosthenuria (1.013).

**Figure 12:**
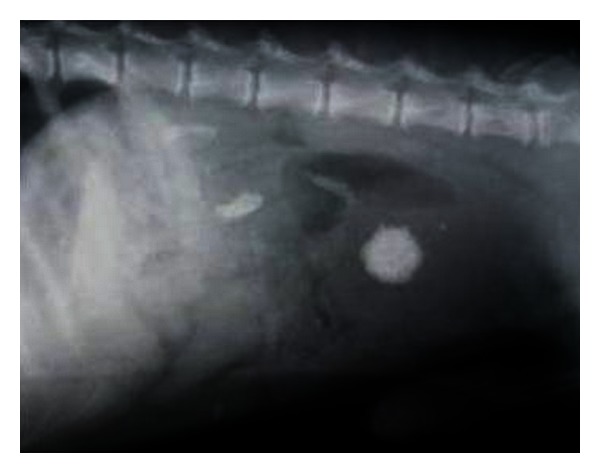
Right lateral survey radiograph depicting radiopaque bilateral nephrolithiasis along with a cystolith in a 9-year old, intact male, Doberman pinscher dog.

**Figure 13:**
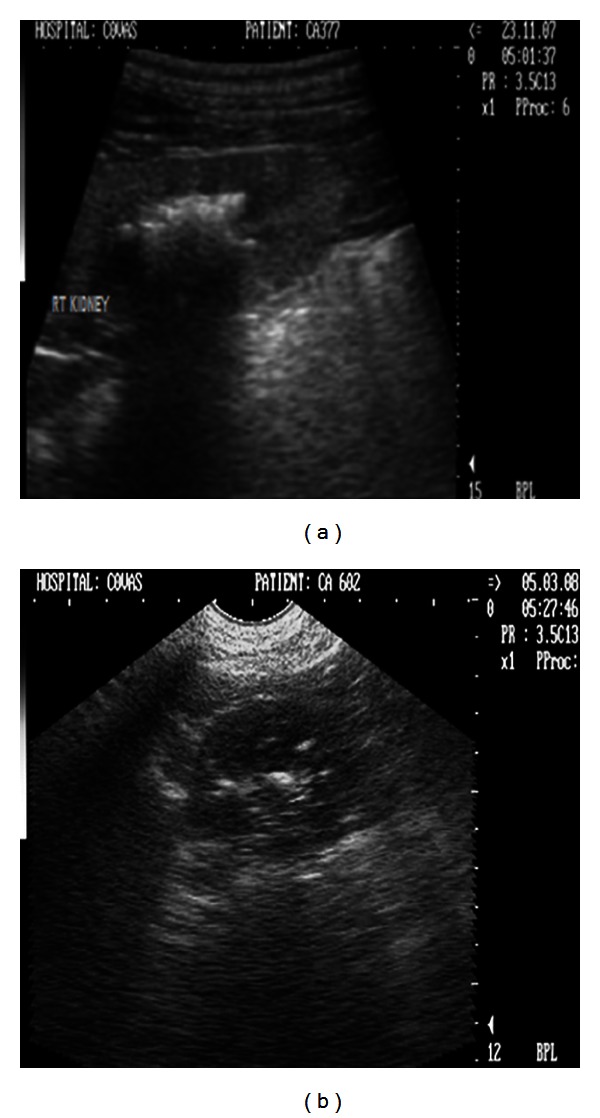
(a) 2D sonogram of right kidney in midsagittal scan revealing large hyperechoic structure in the renal pelvis with intense distal acoustic shadowing and masking of the far calculi renal cortex in a 9-year-old, intact male, Doberman pinscher dog. (b) 2D sonograph (transverse scan) of right kidney revealing scattered hyperechoic densities with acoustic shadowing suggesting renal medullary mineralization in a six-year-old, intact female, mixed breed dog.

 Abdominal lateral survey radiograph revealed two irregular radiopaque densities in the renal regions corresponding in shape with the renal pelvis (Figure 12). Ultrasonographic examination revealed bilateral large hyperechoic structures in the renal pelvis with intense distal acoustic shadowing and masking of the far calculi renal cortex (Figure 13). Besides, the hyperechoic medulla with loss of corticomedullary definition was also observed in both the kidneys. The dog, although on aggressive treatment, died after 7 days of presentation in the clinics.

### 3.6. Nephrocalcinosis

Nephrocalcinosis was diagnosed in a six-year old, intact female, mixed breed dog with body weight of 16 kg. The dog had history of inappetance and vomition. It had been bitten by stray dogs one-week before. Physical examination suggested pallor mucous membrane and deep septic wound on the lateral aspect of right forelimb. Haemogram was unremarkable but the biochemical profile of the dog demonstrated mild azotemia and hypercalcaemia [2].

 Radiographic survey examination was nondiagnostic. Ultrasonographic examination revealed diffuse, small, multiple hyperechoic structures in the renal parenchyma with distal acoustic shadowing (Figure 13). A fine hyperechoic medullary rim was also observed on the left kidney. The dog was rendered conservative medical treatment and recovered well after 7 days of treatment. The dog is fine till date with comfortable haemato-biochemical profile.

### 3.7. Renal Cyst

**Figure 14:**
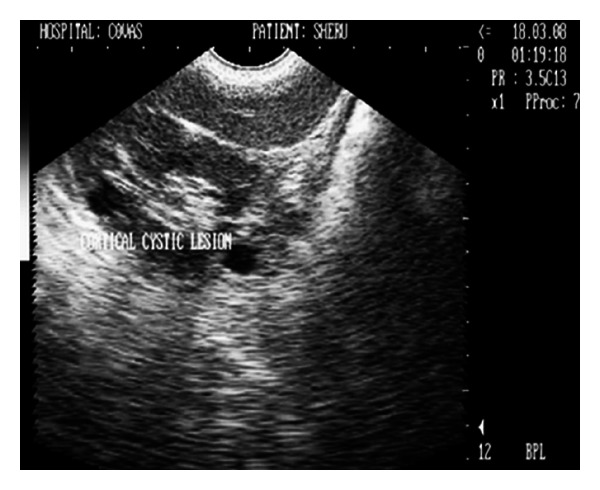
Right kidney sonogram (2D) in sagittal plane revealing two spherical cortical cystic lesions with anechoic fluid in a 16-year-old, neutered male, mixed breed dog.

Two solitary cortical renal cysts were diagnosed in a 16-year-old (geriatric) male dog with body weight of 13 kg. The dog was emaciated with inappetance for the last 2 months. The biochemical profile although in normal range had BUN and creatinine values on higher side. Urinalysis and urine sediment examination was unremarkable. No abnormality was evident on abdominal lateral survey radiograph. Ultrasonographic examination besides having hyperechoic medullary region revealed two small spherical structures in the right renal cortical region which contained anechoic fluid with distal acoustic enhancement (Figure 14). The dog recovered well after conservative medical treatment, that is, fluid therapy, antibacterials, and inj. Ranitidine.

## 4. Discussion

### 4.1. “End-Stage” Kidneys

“End-stage” kidneys and associated CRF were diagnosed in four dogs. Anorexia, progressive lethargy, and weight loss observed in dogs with “end-stage” kidneys may be attributed to accumulation of nitrogenous wastes, metabolic acidosis and changes in the gastrointestinal tract [3]. Vomition in such dogs results as the uraemic toxins act centrally to stimulate the chemoreceptor trigger zone, which, in turn, stimulate the vomiting center [2]. Polyuria and secondary polydipsia observed by the owners in their dogs occur when the renal disease has progressed severe enough to render 66–75% of the nephrons nonfunctional thus compromising the urine concentrating ability [1]. Markedly arched back in the female dog and pain on abdominal palpation may be due to associated renal pathology. Gingivitis, glossitis, and acidotic odour observed in all the dogs may result from alterations in oral flora caused by the local presence of uraemic toxins, which serve as substrate for ammoniagenesis in urease-producing bacteria [3]. Anaemia in all the dogs may be due to chronic renal disease which results from decreased production of erythropoietin and depressed erythrogenesis [4] and/or shortened erythrocyte life span due to accumulation of uraemic toxins [3]. Renal failure results in the retention of uremic toxins and gastrin that damage the gastric mucosa and blood vessels of the gastric wall and result in increased acid production. Ranitidine has been used as gastroprotective drug. Anabolic steroids, Nandrolone, have been administered to stimulate RBC production in anemic animals.

Decreased leukogram, characterized by lymphocytosis and nonregenerative anaemia in one dog, reflected the immunocompromised status of animal. Severe azotemia observed in all the dogs is the result of compromised excretory function where more than 75% of the nephrons are rendered nonfunctional [1]. Hyperphosphatemia and hyperkalaemia observed in the present study in three dogs is the most consistent electrolyte disturbance in patients with renal failure [1]. Isosthenuria with significant azotaemia observed in the present study related with malfunctioning of more than 2/3rd of the nephrons [4, 5] and indicated intrinsic renal failure. Isosthenuria in one dog may have predisposed to the development of urinary tract infection. Survey radiographic visualization failure may be attributed to lack of retroperitoneal contrast due to emaciation as most of the animals were in poor body condition [6]. The failure to visualize the nephrogram and pyelogram following intravenous pyelography in one dog showed the impaired renal excretory function [7]. They reported such visualization failure in animals with “end-stage” kidneys and associated chronic renal failure which is attributed to impaired glomerular filtration and tubular concentrating ability in chronic renal failure. Contrast studies were avoided in other dogs because of poor body condition of the animals. The sonographic observations of decreased size of kidneys could be related to chronic renal failure [8], and the loss of architectural details has been a significant feature of renal diseases due to gradual loss of functional nephrons, over a few months to several years [9]. Complete loss of corticomedullary definition observed in the present study might be due to chronic interstitial nephritis which is a potential cause of “end-stage” kidneys with sonographic observations of small sized kidneys and loss of corticomedullary detail which makes their differentiation from surrounding tissue difficult [10]. In the present study, clinicopathologic manifestation of renal disease and loss of corticomedullary definition on ultrasonographic examination diagnosed the “end-stage” kidneys, though the ultrasonographic features are not specific, and renal biopsy findings are confirmatory for the diagnosis of specific nephropathy.

### 4.2. Hydronephrosis

Chronic renal failure secondary to hydronephrosis following urethrolithiasis was diagnosed in a dog. Retrograde catheterization in this dog confirmed the urethral obstruction which subsequently led to the distention of the urinary bladder and hydronephrosis. The obvious uraemic signs particularly the acidotic odour, glossitis with necrosis of distal part of tongue, and severe azotaemia suggested the associated nephropathy. Biochemical findings of marked azotaemia, hyperphosphatemia, and hyperkalaemia observed in the present study is a feature during oliguria and anuria of terminal CRF [1]. Hypersthenuria with specific gravity of ≥1.030 in the present study might be a result of dehydration following uraemia. Radiography provided information of enlarged kidneys and two small radiopaque calculi in pneumocystograph which otherwise of urethral origin were pushed into the urinary bladder. Sonography demonstrated enlarged kidneys with renal pelvic dilation, separation of the central renal sinus with anechoic space, atrophy of renal medulla, and very classic bilateral hyperechoic medullary rim and moderately dilated ureters as the features of hydronephrosis. The sonographic features like hyperechoic medullary rim are suggestive of nephropathy [11] and marked renal pelvic dilation with central anechoic space and atrophy of renal parenchyma following longstanding ureteral obstruction [10, 12] in hydronephrosis have been documented. Hydronephrosis have also been sonographically categorized as narrowed renal parenchyma with its structures still discernible in mild hydronephrosis to more thin homogenous renal parenchyma in advanced hydronephrosis [13]. According to this classification, the dog in the present study was affected with advanced degree of hydronephrosis. Ureteral obstruction due to calculi, UB tumour, ectopic ureter, and accidental ligation of ureter during ovariohysterectomy has commonly been documented as causes of hydronephrosis. However, in the present study, hydronephrosis was observed secondary to urethrolithiasis. Ultrasonography in the present study proved handy in detecting the parenchymal alterations and avoided the use of more laborious, time consuming excretory urography in animal with poor body condition. Renal biopsy was however recommended for ascertaining the nephropathy.

### 4.3. Renomegaly

Renomegaly and associated nephritis leading to irreversible renal failure was diagnosed in a two-year-old, intact male, German shepherd dog. Survey radiographic failure to visualize the kidneys may again be due to lack of retroperitoneal contrast due to emaciation [6, 14], as the dog had lost substantial body weight before presentation and had poor body status. Sonograph demonstrated enlarged kidneys (left and right kidneys measuring 103 and 98 mm in length and 60 and 62 mm in width, resp.) with increased overall echogenicity and thickness of renal cortex. The maximum value of kidney length in dogs weighing between 25 and 29 kg has been reported to be 78 mm with average value of 69 mm [15]. The observed renal length and width in the present study were substantially higher thus proving renomegaly with associated nephritis. The increased overall echogenicity particularly of renal medullary pyramids, thickness of renal cortex with poor corticomedullary definition as the sonographic features have also been reported in dogs affected with chronic nephritis [11].

### 4.4. Nephritis

Chronic nephritis and associated chronic renal failure (CRF) were diagnosed in an intact female, Pomerarian dog. Ultrasonographic findings of bilateral markedly increased echogenicity of the renal cortex comparable with spleen and fairly defined corticomedullary junction observed in the present study have also been described in chronic nephritis as increased echogenicity of the renal cortex, rarely of the renal medulla; with normal size in early stage to loss of corticomedullary definition with increased overall echogenicity and larger or smaller size in advanced stage [11]. The distended gall bladder and hypoechoic hepatic parenchyma with occasional hyperechoic foci also suggested accompanied hepatitis in the affected dog.

### 4.5. Nephrolithiasis

Nephrolithiasis (bilateral) with associated nephritis and cystolithiasis was diagnosed in a 9-year-old, intact male, Doberman pinscher dog. The clinical signs observed were compatible with uraemia. Flank pain observed in the present case may be attributed to presence of nephroliths. Neutrophilic leucocytosis observed may be due the infectious nephritis as the nephroliths predisposes to bacterial infection by serving as a nidus [16] or may be the result of inflammatory condition of liver as the liver panel was also altered. Laboratory findings of severe azotaemia and isosthenuria (1.013) were in agreement with those reported by Bush [5] and may be attributed to nephrolith induced nephropathy. The irregular radiopaque densities in the renal regions corresponding to the shape of the renal pelvis observed in the present study have been referred as “stag-horn” calculi [14]. Ultrasonographic observations of highly reflective structures in the renal pelvis with intense distal acoustic shadowing and masking of the far calculi renal cortex with medullary compression have been well described [11, 17]. In the present study, however difficulty was encountered while imaging the highly reflective large calculi as it did not allow detecting the true echogenicity of the renal cortex and medulla, the extent of medullary compression and evaluation of most of the renal parenchyma.

### 4.6. Nephrocalcinosis

Nephrocalcinosis was diagnosed in a six-year-old, intact female dog. Inappetance, vomition, anaemia, and mild azotemia observed in the present dog indicated although nonspecifically towards renal pathology. Hypercalcaemia observed might be due merely to an increase in the amount of biologically inactive complexed calcium bound to phosphates, oxalates, and various other anions in their blood which are found in renal failure patients with hyperplastic parathyroid glands with altered set point for detection and response to blood ionized calcium [1]. Radiographic failure in visualization and ultrasonographic findings of diffuse, small, multiple hyperechoic structures in the renal parenchyma with distal acoustic shadowing in the present study have been well documented as focal or diffuse hyperechoic areas with or without distal acoustic shadowing which may be recognized ultrasonographically before being visualized radiographically [17]. A fine hyperechoic medullary rim observed on the left kidney is a unique sonographic feature of nephropathy [11]. The ultrasonographic characterization, strengthened by the haematobiochemical findings in the present study was suggestive of nephrocalcinosis. Sonography in the present study diagnosed nephrocalcinosis in the early stage without specific laboratory indication and radiographic appreciation.

### 4.7. Renal Cyst

Two solitary cortical renal cysts were diagnosed in a 16-year-old (geriatric) male dog. The laboratory data did not provide any indication of pathology. Inappetance and emaciation in the dog under study may be age related. Ultrasonographic findings of two small spherical structures in the right renal cortical region which contained anechoic fluid with distal acoustic enhancement have been well described [17]. The presence of solitary or multiple renal cysts without clinical symptoms are usually of congenital origin and in most cases are incidental findings while carrying out ultrasonographic examination [17].

In conclusion, the affections of kidney, leading to chronic renal failure in majority of dogs were diagnosed in 10 clinical cases. These affections included “end-stage” kidneys (*n* = 4), hydronephrosis (*n* = 1), renomegaly (*n* = 1), nephritis (*n* = 1), nephrolithiasis (*n* = 1), nephrocalcinosis (*n* = 1), and renal cyst (*n* = 1). The significant ultrasonographic features included small kidneys with loss of corticomedullary demarcation making its differentiation from surrounding tissue difficult (“end-stage” kidneys); increased cortical echogenicity and/or increased overall renal echogenicity (nephritis); dilation of the renal pelvis, separation of the central renal sinus with anechoic space, atrophy of renal medulla, and very classic bilateral hyperechoic medullary rim (hydronephrosis); enlarged kidneys with increased overall echogenicity and thickness of renal cortex (renomegaly and associated nephritis); hyperechoic-mineralized structure with masking of far calculi renal cortex (nephrolithiasis); diffuse, small, multiple hyperechoic structures in the renal parenchyma with distal acoustic shadowing (nephrocalcinosis) and small spherical structures in the renal cortical region containing anechoic fluid with distal acoustic enhancement (renal cysts). In the present study, ultrasound proved to be a quick, convenient and sensitive modality in detecting alterations in renal size and parenchymal architecture. Thus, it overcame many of the shortcomings of abdominal palpation and eliminated the need to perform the more laborious excretory urography and renal biopsy which are generally unnecessary in patients with well-defined historical, clinical, laboratory, and ultrasonographic evidence of renal failure.

## Figures and Tables

**Table 1:** Hematological profile in different affections of kidney.

Disease condition	Hb (g %)	PCV (%)	TEC (×10^12^/L)	TLC (×10^9^/L)	DLC (%)				
					N	L	M	E	B
“End-stage” kidneys (*n* = 4)	10.23 ± 2.38	29.75 ± 7.59	5.8 ± 0.62	11.8 ± 2.66	63 ± 8.09	32.75 ± 8.26	2.5 ± 0.29	1.67 ± 0.29	1 ± 0
Hydronephrosis (*n* = 1)	11	48	6.46	20.85	82	12	4	2	—
Renomegaly (*n* = 1)	12.2	40	6.69	6	69	25	2	2	2
Nephritis (*n* = 1)	14	38	6.34	18.8	42	54	—	4	—
Nephrolithiasis (*n* = 1)	11	47	7.12	12.95	78	20	2	—	—
Nephrocalcinosis (*n* = 1)	8.5	28	6.82	11.2	67	28	2	2	1
Renal cyst (*n* = 1)	9.5	36	6.02	7.3	64	28	2	4	2

**Table 2:** Biochemical profile in different affections of kidney.

Disease condition	ALT (U/L)	AST (U/L)	Total bilirubin (mg/dL)	BUN (mg/dL)	CRTN (mg/dL)	Total protein (g/dL)	Glucose (mg/dL)		Electrolyte assay (mmol/L)		
								Sodium	Potassium	Inorganic phosphate	Total calcium
“End-stage” kidneys (*n* = 4)	60.25 ± 22.45	74.5 ± 47.68	1.88 ± 1.69	109 ± 32.05	8.98 ± 2.22	6.68 ± 0.31	74.86 ± 2.04	142 ± 3.23	5.9 ± 0.82	2.02 ± 0.12	2.24 ± 0.02
Hydronephrosis (*n* = 1)	21	18	0.45	156.2	22.9	6.3	78	143	6.01	1.8	2.52
Renomegaly (*n* = 1)	97	71	0.64	183.6	17.5	5.0	71	145	4.8	1.6	2.25
Nephritis (*n* = 1)	90	134	0.73	146.7	13.0	8.6	82	147	5.2	1.53	2.34
Nephrolithiasis (*n* = 1)	88	70	1.4	128	6.12	6.0	76	153	5.6	1.48	2.22
Nephrocalcinosis	27	23	0.6	68.4	2.3	5.8	73	154	4.6	0.9	3.24
Renal cyst (*n* = 1)	19	27	0.63	78	2.1	6.4	85	148	4.9	1.7	2.43

**Table 3:** Dipstick urine analysis of the different affections of kidney.

Test	Renal affections						
	“End-stage” kidneys	Hydronephrosis	Renomegaly	Nephritis	Nephrolithiasis	Nephrocalcinosis	Renal cyst
Colour	Clear	Brownish red	Clear	Clear	Clear	Clear	Straw coloured
Specific gravity	1.007–1.012	≥1.030	1.012	1.014	1.013	1.016	1.021
pH	6.9–7.8	6.0	7.6	7.4	7.2	6.8	6.2
Protein (mg/L)	150–300	100	225	100	250	200	−ve
Glucose (mg/dL)	200–350	100 mg/dL	75	50	225	25	−ve
Ketone	Negative	Negative	Negative	Negative	Negative	Negative	Negative
Bilirubin	Negative	Negative	Negative	Negative	Negative	Negative	Negative
Blood	Negative	50 RBCs/*μ*L	Negative	Negative	Negative	Negative	Negative
Urobilinogen	Negative	Normal	Normal	Normal	Normal	Normal	Normal

**Table 4:** Urine sediment examination results in different affections of kidney.

Element	Renal affections						
	“End-stage” kidneys	Hydronephrosis	Renomegaly	Nephritis	Nephrolithiasis	Nephrocalcinosis	Renal cyst
Epithelial cells	1-2 per hpf	Very few, transitional	0–2 per hpf	3–5 per hpf	2–5 per hpf	1-2 per hpf	0–2 per hpf
Crystals	Oxalates and triple phosphate crystals	Few, triple phosphate	Negative	Negative	Calcium oxalate monohydrate crystals	Few, calcium phosphate	Bilirubin crystals
RBC	2–4 per hpf	Too numerous to count	None	None	6–8 per hpf	None	None
WBC	4–6 per hpf	15–30 per hpf	2-3 per hpf	3–5 per hpf	4–6 per hpf	Negative	Negative
Debris	None	Large amount	None	None	Very few	None	None
Casts	Few hyaline casts	Occasional epithelioids with white and red cell casts	Epithelial cell casts	Hyaline (very rare)	Few, Granular casts	None	None
